# Bayesian Learning Aided Theoretical Optimization of IrPdPtRhRu High Entropy Alloy Catalysts for the Hydrogen Evolution Reaction

**DOI:** 10.1002/smtd.202401224

**Published:** 2024-11-10

**Authors:** Linke Huang, Zachary Gariepy, Ethan Halpren, Li Du, Chung Hsuan Shan, Chuncheng Yang, Zhi Wen Chen, Chandra Veer Singh

**Affiliations:** ^1^ Department of Materials Science & Engineering University of Toronto 184 College Street, Suite 140 Toronto ON M5S 3E4 Canada; ^2^ Key Laboratory of Automobile Materials Ministry of Education and School of Materials Science and Engineering Jilin University Changchun 130022 P. R. China; ^3^ Department of Mechanical & Industrial Engineering University of Toronto 5 King's College Road Toronto ON M5S 3G8 Canada

**Keywords:** Bayesian Learning, cost reduction, high entropy alloys catalyst, hydrogen evolution reaction, theoretical composition optimization

## Abstract

The complex compositional space of high entropy alloys (HEAs) has shown a great potential to reduce the cost and further increase the catalytic activity for hydrogen evolution reaction (HER) by compositional optimization. Without uncovering the specifics of the HER mechanism on a given HEA surface, it is unfeasible to apply compositional modifications to enhance the performance and save costs. In this work, a combination of density functional theory and Bayesian machine learning is used to demonstrate the unique catalytic mechanism of IrPdPtRhRu HEA catalysts for HER. At high coverage of underpotential‐deposited hydrogen, a *d*‐band investigation of the active sites of the HEA surface is conducted to elucidate the superior catalytic performance through electronic interactions between elements. At low coverage, a novel Bayesian learning with oversampling approach is then outlined to optimize the HEA composition for performance improvement and cost reduction. This approach proves more efficacious and efficient and yields higher‐quality structures with less training set bias compared with neural‐network optimization. The proposed HEA optimization theoretically outperforms benchmark Pt catalysts’ overpotential by ≈40% at a 15% reduced synthesis cost comparing to the equiatomic ratio HEA.

## Graphical abstract

High‐entropy alloys (HEAs) can offer cost‐effective and enhanced catalytic performance for hydrogen evolution reaction (HER) through compositional optimization. This study combines density functional theory and Bayesian machine learning to elucidate unique catalytic mechanisms of IrPdPtRhRu HEA. A novel Bayesian approach improves composition optimization, achieving superior catalytic efficiency and reducing synthesis costs compared to equiatomic HEAs and Pt benchmarks.

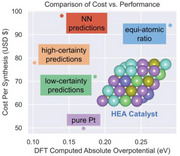

## 1 Introduction

Global awareness of climate change has been steadily rising for decades due to social pressure and devastating potential health effects on humans.^[^ ^1^, ^2^, ^3^, ^4^, ^5^ ^]^ Although climate change stems from countless industries and chemical reactions, it is well known the oil/gas industry paired with the automotive industry are large contributors to global warming due to the polluting byproducts of gas‐powered engines.^[^ ^6^, ^7^ ^]^ Therefore, alternative energy carriers should be explored, especially hydrogen with the highest energy density of 142 MJ kg^−1^.^[^ ^8^, ^9^, ^10^ ^]^ To accelerate the application of hydrogen energy, the most efficient commercial water electrolyzers often employ a polymer exchange membrane to conduct protons and pure Pt cathodes to catalyze the hydrogen evolution reaction (HER).^[^ ^11^, ^12^, ^13^ ^]^ Nevertheless, the widespread adoption of Pt is impeded by its exorbitant cost and limited availability, obstructing its viability for large‐scale applications. Thus, the imperative lies in crafting high‐performance, economically viable Pt‐based catalysts to propel the advancement of a hydrogen economy.

Recent innovations in HER catalysts have shown that more efficient conversion can be obtained through multi‐component alloy catalysts such as high entropy alloys (HEAs).^[^ ^14^, ^15^, ^16^ ^]^ Noble metal HEAs exhibit superior catalytic activity due to their high intrinsic activity and stability.^[^ ^17^, ^18^, ^19^ ^]^ On the other hand, HEAs with a combination of noble and non‐noble metal and pure non‐noble metal have gained attention for their cost‐effectiveness and comparable catalytic performance in certain reactions.^[^ ^20^, ^21^, ^22^, ^23^ ^]^ In particular, the Kitagawa group experimentally synthesized an IrPdPtRhRu HEA nanoparticle (NP), which showed a better catalytic performance (activity and stability) compared with traditional Pt catalysts.^[^ ^15^ ^]^ However, the application of HEA catalysts is still in its nascent stages. The vast compositional space and structural complexity present significant challenges for further exploration in the realm of HEA catalysts.^[^ ^24^ ^]^ Most computational studies leverage density functional theory (DFT)‐informed machine learning (ML) models to discern trends and optimize specific material properties.^[^ ^24^, ^25^, ^26^, ^27^, ^28^, ^29^, ^30^, ^31^ ^]^ However, a potential limitation of these approaches is that the atomic models are often constrained to the nearest‐neighbor scale, offering limited variation in the local chemical environment due to the periodic boundary conditions. This can reduce their direct applicability to experimental settings. Furthermore, while some research groups have focused on optimizing compositions, they frequently neglect the economic aspects of HEA synthesis. This oversight significantly contributes to the limited commercial viability of technologies such as proton exchange membrane water electrolyzers and fuel cells.^[^ ^13^, ^14^ ^]^ Therefore, compositional modifications should be implemented to maximize catalytic performance whilst minimizing cost. This would represent a step in the direction towards making hydrogen fuel for automobiles competitive with gas engines or Li battery stacks.^[^ ^11^, ^12^, ^13^ ^]^ Moreover, industry viability, scalability, and cost need to be considered to achieve disruption in the automotive industry.

In this work, the HEA system of IrPdPtRhRu was selected as our model system. Through DFT simulations, we unravel the mechanism of HER, shedding light on the crucial role of surface coverage in determining HER performance. Furthermore, a novel Bayesian ML and oversampling technique is presented to optimize the HEA composition, which resulted in a 40% superior theoretical overpotential compared to Pt, a 15% reduction in cost, and improved resistance to raw resource market volatility. The novel optimization method was found to be 400% more efficient in compositional exploration with a deeper and less biased understanding of ideal catalyst surfaces compared to traditional non‐Bayesian ML methods.

## 2 Results and Discussion

For efficient catalysis of the HER, it is important to consider the unique surface effects that occur on certain platinum group metals (PGMs).^[^ ^32^, ^33^, ^34^, ^35^, ^36^ ^]^ The impact of H* coverage (θ_H_) has been extensively explored in the literature, with consensus indicating distinct mechanisms between high H* coverage (θ_H_ ≈ 0.7–1.0) and low coverage (θ_H_ ≈ 0.0–0.4).^[^ ^34^, ^36^ ^]^ At higher coverage, underpotential‐deposited hydrogen (H_UPD_) in the FCC hollow sites is strongly bound and inactive. Consequently, overpotential‐deposited hydrogen (H_OPD_) in the top sites becomes the active hydrogen atoms.^[^ ^34^, ^36^ ^]^ At lower coverage, the electronic structure of the surface is different due to lower electron counts originating from the absence of H_UPD_ atoms. To fully explore the reaction mechanism of HER on HEA systems, we investigated these two cases separately.

### 2.1 High Surface Coverage

#### 2.1.1 Theoretical Explanation of IrPdPtRhRu Catalytic Superiority

**Figure 1:**
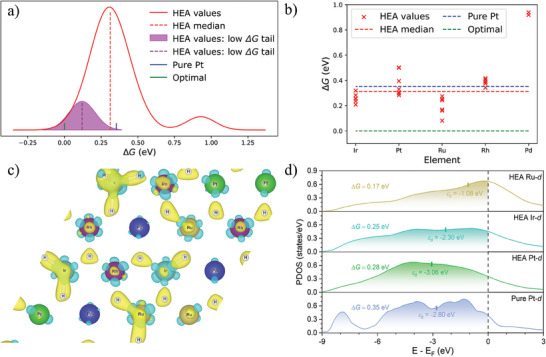
a) The distribution of Δ*G_H_* values of $\frac{1}{16}\;ML$ of H_OPD_ on IrPdPtRhRu (111) and pure Pt (111) containing 1ML of H_UPD_ in the FCC hollow sites. b) The Δ*G_H_* values for each top site element of the HEA. The charge density difference of 1 monolayer H_UPD_ adsorbed on c) IrPdPtRhRu and the yellow isosurface represents electron accumulation and the cyan isosurface represents electron depletion with an isosurface level of 0.01. d) The projected density of states (PDOS) for three superior HEA top sites and the top site of pure Pt. As seen in inset (b), Ru is the optimal top site in high coverage scenarios, with Ir, Pt, and Rh also offering adsorption sites preferable to pure Pt. Although Ru bonds stronger than Ir to H_OPD_, the inverse case is true for H_UPD_, as displayed in inset (c). The repulsion between H_UPD_ and H_OPD_ atoms is a significant contributor to the weak bonding of H_OPD_ to the top sites, but this effect is alleviated by increasing the physical distance between H_UPD_ and H_OPD_. This result shows how noble metal surfaces can be engineered to create more optimal active sites for the H_OPD_ intermediate based on atomic radii. Embedding atoms with larger radii into noble metal surfaces can be a strategy that should be further explored for cases where there is a high coverage of H_UPD_.

Slab models (Figure S1, Supporting Information) of IrPdPtRhRu(111) and pure Pt(111) were first generated. A full monolayer of H_UPD_ on the FCC hollow sites was included to represent the maximum scenario of H_UPD_ coverage as a simplification to have a uniform electronic environment for H_OPD_ adsorption, as displayed in Figure S2 (Supporting Information). The adsorption energy of H_OPD_ at top sites was computed for a coverage of $\frac{1}{16}$ monolayer of H_OPD_ with the presence of a full monolayer of H_UPD_. Unlike the state‐of‐the‐art catalyst of Pt, which typically exhibits a uniform adsorption energy value, the IrPdPtRhRu surfaces displayed a distribution of Δ*G_H_* values due to the unique local chemical environments present at each top site. On pure Pt (111), the H_OPD_ has a computed hydrogen‐adsorption free energy (Δ*G_H_*) =  0.35 *eV*, which is in good agreement with values reported by Santos et al.^[^ ^37^ ^]^ The distribution of computed values for Δ*G_H_* of H_OPD_ is shown in **Figure** **1**a. The HEA top sites provide a broad distribution of adsorption free energy near 0 eV, encompassing a more extensive array of optimal active sites. The negative tail of the distribution with median value lower than the overall distribution will be the major adsorption sites of H* during HER (denoted as low Δ*G* tail in Figure 1a).^[^ ^16^, ^38^ ^]^ In contrast, pure Pt surface lacks such a distribution of Δ*G_H_* values, and thus does not exhibit this beneficial effect. Consequently, the substantial improvement in the median Δ*G_H_* between pure Pt and the low Δ*G* tail of IrPdPtRhRu HEA provides a compelling explanation for the HEA's superior catalytic performance.

#### 2.1.2 Insights into the Electronic Structure of IrPdPtRhRu

The charge density differences were explored to understand the trends in bond strength between the H_OPD_ of IrPdPtRhRu and Pt surfaces. As seen in Figure 1c, H_UPD_ atoms are bonded the strongest to Ir, with Ru possessing the second strongest bond. The Pd atoms don't participate in bonding, likely because they possess a filled d‐state. The Ir and Ru atoms have three unfilled 5*d* orbitals and three unfilled 4*d* orbitals, respectively, the most unfilled *d*‐orbitals of the available atoms. Despite a bonding stronger to H_UPD_, the Ir top sites did not bond as strongly to H_OPD_ as the Ru top sites. This result supports the theory that atomic radius can be used to minimize coulombic repulsion between H_OPD_ and H_UPD_ atoms. For all Ru top sites, there was a correlation between the Δ*G_H_* of H_UPD_ and the Δ*G* of H_OPD_. The more electrons the Ru atom transferred to H_UPD_, the stronger it bonded to H_OPD_. This is possibly due to the interaction with H_UPD_ causing the *d*‐band to shift upwards. Figure S3 (Supporting Information) shows how H_UPD_ bonding on pure Pt is symmetrical with minimal charge redistribution in contrast.

Santos et al. explained that, in addition to Δ*G_H_* ≈ 0 eV, a good HER catalyst will also have a *d*‐band that spans across the Fermi level.^[^ ^37^ ^]^ This contributes to lowering the activation energy of H*. The electronic structure of three top sites from IrPdPtRhRu was compared with the pure Ru, Ir, and Pt top site, shown in Figure 1d and Figure S4 (Supporting Information). The projected density of states (PDOS) are calculated for the FCC(111) surfaces (HCP(0001) for pure Ru) with a monolayer of H_UPD_ in the FCC hollow sites before the H_OPD_ was adsorbed. The results suggested that the d‐band center of Ru, Ir, and Pt sites in the HEA follows the expected trend of pure metals and a correlation exists between higher *d*‐band centers and stronger adsorption energies within the HEA, consistent with traditional *d*‐band theory. Notably, while pure Ru has a *d*‐band center closest to the Fermi level compared to Pt and Ir, the adsorption behavior does not strictly follow trend. Ru exhibits the strongest adsorption at the FCC site but the weakest at the atop site (Table S1, Supporting Information), highlighting that the adsorption behavior in HEA sites is not solely determined by individual elements but also influenced by the synergistic interactions among them. Furthermore, the lower Δ*G_H_* reaction sites in the HEA exhibit stronger hybridization of anti‐bonding states between the H 1s and metal d orbitals, while the superior sites have d‐bands that span more of the Fermi level, enhancing their activity.^[^ ^29^ ^]^

### 2.2 Low Surface Coverage

#### 2.2.1 Database Generation and Machine Learning Modeling

**Figure 2:**
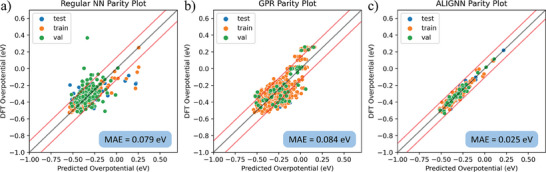
a) Parity plot of the NN used for benchmark comparison. b) Parity plot of the GPR with SMOGN oversampling. c) Parity plot of the ALIGNN model. All outer red lines represent 1 standard deviation of the predicted MAE scores for each model.

The workflow of Section 2.2 is visualized as shown in Figure S5 (Supporting Information). To study the active hydrogen species contributing to the HER at low surface coverage, a database with 800 adsorption models of $\frac{1}{16}$ monolayer of H_OPD_ at top site is generated for ML modeling, composition and cost analysis, and optimization. The average DFT computed Δ*G_H_* of the database across all surfaces was −0.321 with a 0.114 eV standard deviation. For machine learning analysis, the 15 nearest atoms to the hydrogen adsorbate were extracted and over 60 unique numeric atomic descriptors were initially evaluated and screened. Three ML models were trained on the database: a regular neural network (NN) for benchmark comparisons, a Bayesian Gaussian process regressor (GPR) for novel optimization methodologies, and a graph neural network (GNN) for maximum modeling accuracy. More details of model design and feature engineering are shown in Section SII (Supporting Information). The benchmark NN model obtained a mean absolute error (MAE) accuracy of 0.079 eV but struggled to accurately predict the rare data region between –0.050–0.200 eV. By applying the Synthetic Minority Over‐sampling with Gaussian Noise (SMOGN) technique developed by Branco et al., the dataset size was increased by ∼50 data points (Figure S7, Supporting Information)^[^ ^39^ ^]^ and the GPR model obtained an MAE score of 0.084 eV with improved predictive capabilities of low represented Δ*G_H_* regions (**Figure** **2**a,b). Since the least represented Δ*G_H_* regions were −0.05–0.2 eV, the over‐sampling technique improved the Bayesian model's ability to predict optimal Δ*G_H_* configurations, while retaining a comparable accuracy against the benchmark NN. Applying the Atomistic Line Graph Neural Network (ALIGNN) model developed by K. Choudhary,^[^ ^40^ ^]^ an average MAE value of 0.025 eV was obtained (Figure 2c). The superior accuracy can be attributed to graph‐based models’ ability to capture larger degrees of spatial and angular information due to the inherent nature of node and edge‐based feature design relative to array‐based feature design. Unlike the NN and ALIGNN model, the GPR oversampling created three distinct clusters visible in the parity plots. This is due to the data points in rarer regions that created clusters of additional data points in the 0, 0.1, and 0.3 eV ranges (Figure 2b). Comparing to ALIGNN which required the information of the whole slab model as input, sacrificing accuracy for optimizing viable feature sets was necessary for the GPR and NN models. Therefore, graph‐based models were not utilized to reduce the computational cost for further compositional space exploration and optimization study.

#### 2.2.2 Composition Optimization of HEA by Machine Learning

**Figure 3:**
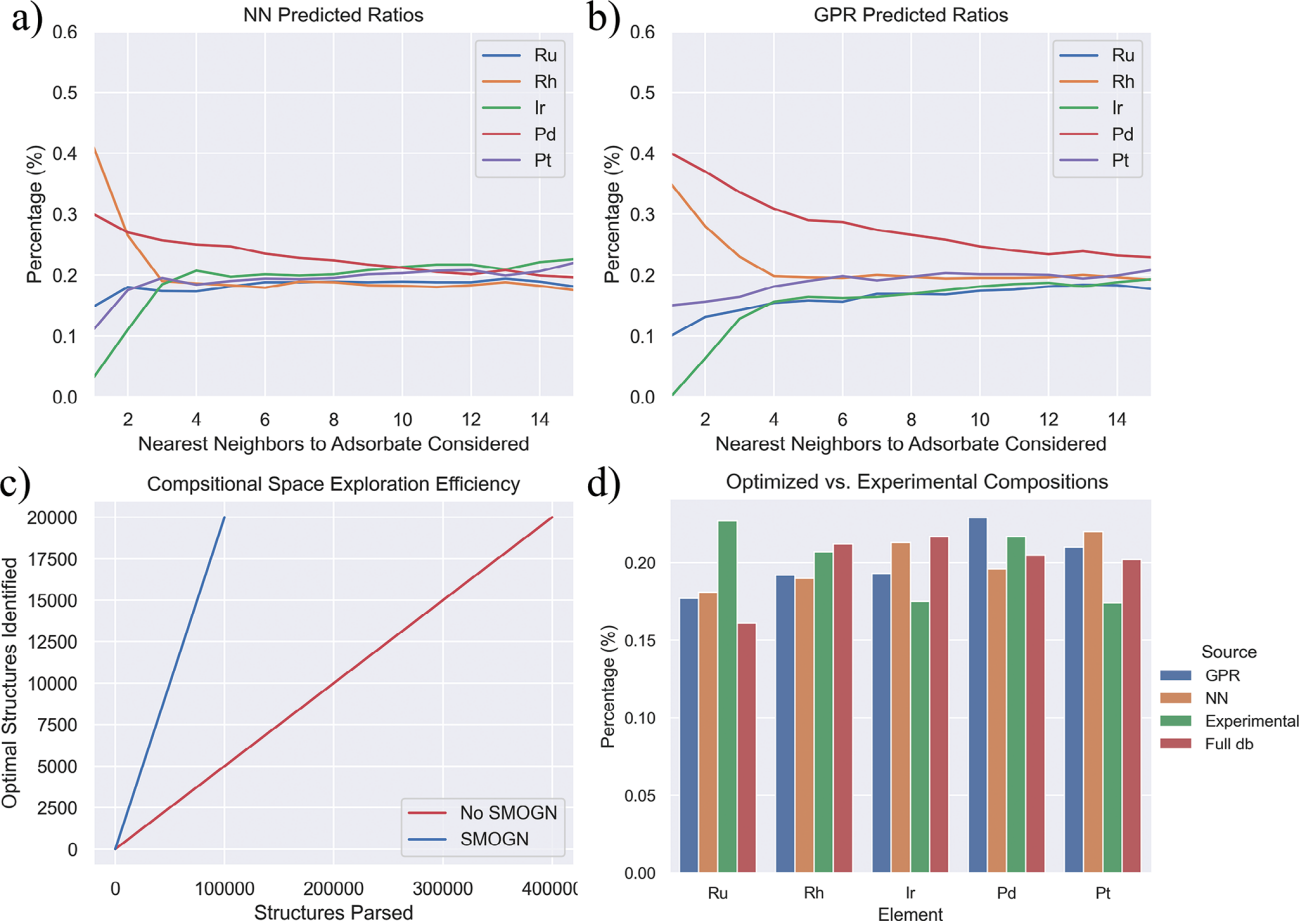
a) Average NN predicted composition of optimized surfaces based on proximity to adsorbate. b) Average GPR predicted composition of optimized surfaces based on proximity to adsorbate. c) Surface discovery efficiency of a SMOGN‐based GPR model and a baseline NN model. d) NN and GPR optimized surface compositions compared to the un‐optimized database and experimental surface composition obtained by XRF.^[^ ^15^ ^]^

To investigate the composition space of the IrPdPtRhRu HEA, we initially examine the efficacy of NN and GPR models in identifying local chemical environments that can have a more optimal overpotential by populating the confusion matrix. The *∆G*_H_ distribution with the variation of each metal component for the DFT‐calculated adsorption database is visualized in Figure S8 (Supporting Information). The data show fewer samples at extreme low (<6.7%) and high (>33.3%) compositions, suggesting potential sampling bias. Consequently, the correlation between metal composition variation and HER performance (*∆G*_H_) from the database is weak. Therefore, we focused on analyzing compositions predicted by ML models to have more promising performance. The NN and GPR models exhibit a sensitivity of 63% and 68%, respectively at a cutoff overpotential of |0.17 V|, and sensitivity of 36% and 50% with a more stringent cutoff of |0.09 V|. The abnormal increase of the sensitivity with reducing cutoff might be caused by sampling bias, but GPR overall shows a stronger ability to capture optimal adsorption sites. The two models were then tasked with predicting random compositions of the 15 nearest atoms to the hydrogen adsorbate sorted by distance (Section SIIC, Supporting Information) to identify 20 000 unique structures with desirable predicted overpotentials (< |0.09 V|). Comparing the structures predicted by the NN and GPR, the application of SMOGN to the Bayesian model offered two benefits not seen in the benchmark ML optimization. First, composition exploration was 400% more efficient compared to the NN exploration (**Figure** **3**c). To discover 20 000 unique 15‐atom local chemical environments with expected overpotentials close to 0 (−0.09 to 0.09 V), the GPR model parsed 100 000 datapoints whereas the NN parsed 400 000 datapoints. Secondly, the GPR model achieved a higher compositional variety with a more balanced distribution of Rh, Pd, and Pt‐top site structures, arising from the application of SMOGN oversampling technique. The optimized composition proposed by GPR model displayed a reduced training set bias correlated to the trend of the original database compared to the NN model, which exhibited a strong preference for Rh‐top sites. As seen in Figure 3a,b, both models tend to predict a higher percentage of Pd and Rh with decreasing cutoff distance from the adsorbate, the GPR displayed smaller bias towards Rh. For Ir, the NN and Bayesian optimizations contained larger proportions compared to experimental surfaces but almost 0 Ir binding sites. This suggests Ir is beneficial as a neighbor atom for HER but a suboptimal binding site location. These findings suggest that our GPR model provided an effective broadening of the compositional search space beyond the limitations observed in the original database. Comparative analysis with experimental compositions from XRF by the Kitagawa group, the original computed database, and the NN & GPR model predictions revealed key insights into the elemental distribution optimal for HER catalysis.^[^ ^15^ ^]^ While the optimized structures by both models maintained similar Ru percentages to the database, they contained ≈5% less Ru and an increased composition of Pd and Pt than the experimental measurement, indicating a potential area for further optimization (Figure 3d). The difference between the ML‐optimized compositions and the DFT study of high‐coverage study in Section 2.1 is attributed to the presence of monolayer H_UPD_ coverage. The H_UPD_ hollow site adsorption resulted in a different electronic surface structure that is not present in the ML training database and thus was not considered in the optimized structures, which indicated the dependence of HEA's catalytic behavior for HER on the surface coverage(θ_H_).

To verify the reliability of the NN and GPR models, 6 GPR‐optimized structures and 3 NN‐optimized structures were extracted and simulated in DFT (Section SIID, Supporting Information). The 6 GPR‐predicted structures possessed desirable ML predicted overpotentials (< |0.09 V|) with Pt or Pd binding sites to ensure the Bayesian model was not overestimating the performance of the rare surfaces in the database. Three of the structures with these criteria were extracted from the top 10% highest certainty predictions and three of the structures were extracted from the bottom 10% lowest certainty structures to be compared and highlight the benefits of utilizing a Bayesian model uncertainty quantification as illustrated in Figure S9 (Supporting Information). Through performing feature space analysis over the original dataset, it is revealed that the high‐certainty structures have a greater average data density around them in comparison to the low‐certainty predictions (See Figure S10, Supporting Information), while the high data‐density region could be inherently correlated to local chemical environments that are more thermodynamically favorable. Such potential difference in reliability is further confirmed by DFT calculation: the high‐certainty predictions possessed a lower MAE than the low‐certainty predictions. The average DFT calculated overpotential of high‐certainty structures was 0.10 V with an MAE of 0.042 V. The low‐certainty structures had an average DFT overpotential of 0.18 V with an MAE of 0.16 V. For the NN‐optimized structures that were randomly selected (2 Pd and 1 Pt binding sites), the average DFT overpotential value was 0.14 V with an MAE of 0.13 V. This result highlights the unique ability of Bayesian models to generate high‐confidence ML‐designed surfaces that outperform traditional NN optimizations. In both optimization applications, a deviation from equiatomic composition suggests the experimental catalytic performance can be improved if minor tweaks to the Kitagawa group's one‐pot polyol process are applied.

#### 2.2.3 Techno‐Economic Analysis

**Figure 4:**
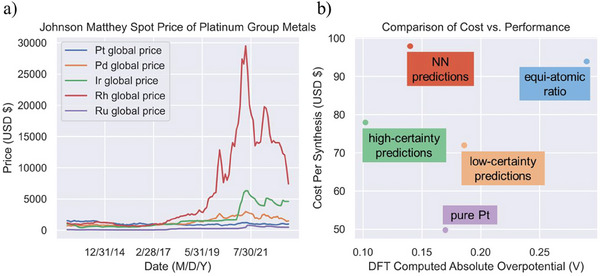
a) Historic value of transition metals from 2013 to present based on Johnson Matthey Spot Pricing as an average value across American, EU, and Asia markets. b) DFT computed overpotential (absolute value) and cost per synthesis (USD) based on chemical precursor pricing as of April 4, 2023 obtained from Fisher Scientific reagents.

The techno‐economic analysis of HEA compositions considered historic metal ore prices, processed precursor reagent pricings, and molar compositions for the synthesis cost and the absolute overpotential for catalytic performance. Historic data on the metal ore costs were obtained from the Johnson Matthey Spot price of each PGM dating back to 2013 for Asian, European (EU), and American markets (Figure S11, Supporting Information) and the average is displayed in **Figure** **4**a.^[^ ^41^ ^]^ It is shown that the price of Rh metal ore is significantly larger than the other four elements. Sharing the same dominant producer (South Africa), the price of Rh displayed a much larger volatility than any other four elements due to the small size of market and its limited production as a byproduct of the mining of Pd, Pt, and Ni. Considering the growing market and industrial demand associated with its application in catalysts for automotive and chemical industries, the price of Rh and its chemical precursor is expected to remain fluctuating in the future. The relative high price and volatility makes Rh the major variable for the synthesis cost of the HEA nanoparticles.

The price of metal reagents was obtained from Sigma Aldridge or Fisher Scientific. The exact metal precursors were selected based on the methods used by the Kitagawa group^[^ ^15^ ^]^ in the one‐pot polyol process (see Figure S12, Supporting Information for details). The cost per synthesis was calculated based on the number of mols in 50 mL, molecular weight, and the cost per gram of precursor used to obtain a specific compositional ratio shown in Equation (1).

(1) $$\sum_{i=1}^{5}\left[mols\;of\;precursor\;i\right]*\left[precursor\;M_{w}\right]*\left[cost\;per\;g\;precursor\right]$$

where *i* is one of five constituent elements of the optimized IrPdPtRhRu HEA (see Figure S13, Supporting Information for sample calculations).

As the baseline data, the experimental synthesis conditions detailed by the Kitagawa group ^[^ ^15^ ^]^ were used which comprises an equimolar ratio of the five metal precursors resulting in an approximate cost per synthesis of $95 USD and the corresponding computational overpotential was calculated as the mean absolute overpotential of 111 surface data points in the database. The cost of synthesizing a pure Pt nanoparticle using the same one‐pot polyol process was $49. The pure Pt datapoint was calculated using the same DFT parameters as the HEA for direct comparison and resulted in a 0.17 V overpotential which aligns with the literature.^[^ ^36^ ^]^ The compositions of “NN‐predictions” and “high/low certainty predictions” refer to the optimized structures predicted by NN and GPR models. The overpotentials are calculated as the average of DFT calculation for the selected structures in Section 2.2.2 and the synthesis cost is calculated according to Equation (1).

Due to the relatively low price of Pt ore and chemical precursors, the pure Pt catalyst remains the cheapest catalyst considered, but is no longer the top‐performing catalyst. The NN‐predicted structures outperformed the pure Pt catalyst, while the high Rh compositions of the surfaces make it the most expensive among all candidates. The high and low‐certainty predictions both displayed ≈15% reduction in the cost of synthesis in comparison to the un‐optimized database (“equiatomic ratio”), and the high‐certainty predictions show 65% theoretical overpotential improvement over equiatomic composition and 40% over the benchmark Pt catalyst.

Based on DFT and ML investigation of high and low θ_H_ environments, the compositional insights derived from this study theoretically improved HER overpotential from 0.29 to 0.10 eV while reducing synthesis costs from ≈$95 to ≈$82 per synthesis with facile scalability and price stability. Experimental adjustments to the one‐pot polyol synthesis methods can be found in **Table** **1** below:

**Table 1:** Experimental parameters and the resulting composition versus recommended adjustments.

Metal	Initial Molarities/Composition [%]	XRF Determined Compositional Variance from initial molarities [%]	GPR recommended initial Composition [%]
Pt	20.00	19.51 ± 0.29	22.50
Pd	20.00	20.74 ± 0.31	24.50
Ir	20.00	19.06 ± 0.40	18.00
Rh	20.00	19.93 ± 0.13	15.00
Ru	20.00	20.75 ± 0.30	20.00

## 3 Conclusion

In the current work, an experimentally synthesized IrPdPtRhRu HEA with exceptional HER catalytic performance was computationally studied to reveal how it breaks *d*‐band theory trends, and an alternative explanation for the HEA's superior performance is proposed. The HEA was then optimized through a novel Bayesian oversampling approach that explored the compositional space of the HEA more efficiently than traditional non‐Bayesian ML models. The framework trained a GPR on an 800 datapoints dataset and obtained an ≈0.084 eV MAE score. The performance‐optimized HEA composition was lastly put through a techno‐economic analysis. By performing a dual cost and performance optimization of the HEA through DFT and Bayesian ML, exact experimental synthesis adjustments were proposed to synthesize IrPdPtRhRu HEA with a theoretical 40% superior overpotential compared to Pt(111) and ≈15% reduced synthesis cost than the equiatomic ratio. This study also provided an analysis of saturated versus non‐saturated θ_H_ environment catalytic influences. The work presented the first‐ever investigation into the benefits of SMOGN oversampling paired with Bayesian learning for catalytic compositional optimization. Future works can focus on further refinement of Bayesian models through the application of Bayesian GNNs and experimental validation of the suggested compositional adjustments.

## Conflict of Interest

The authors declare no conflict of interest.

## Author Contributions

L.H., Z.G., Z.W.C., and C.V.S. conceived and designed the study. Z.G., E.H., and J.S. simulated the VASP dataset. L.H. and Z.G. performed all data analytics and modeling. Advanced DFT analysis was performed by L.H., E.H., and Z.G. The paper was written by L.H., Z.G., E.H., Z.W.C., and C.V.S. All authors discussed and revised the manuscript.

## Supporting information



Supporting Information

## Data Availability

The data that support the findings of this study are available from the corresponding author upon reasonable request.
