# Supplementary material for: Bayesian Learning Aided Theoretical Optimization of IrPdPtRhRu High Entropy Alloy Catalysts for the Hydrogen Evolution Reaction
Source: Small Methods. 2024 Nov 10;9(5):2401224. doi: 10.1002/smtd.202401224 (PMC12103230; doi:10.1002/smtd.202401224)
Supplement: Supplementary file 1 — Supporting Information [file SMTD-9-2401224-s001.docx]

Supplementary Materials for

**Bayesian Learning Aided Theoretical Optimization of IrPdPtRhRu High Entropy Alloy Catalysts for the Hydrogen Evolution Reaction**

Linke Huang^1^ , Zachary Gariepy^1^ , Ethan Halpren^1^ , Li Du,^2^ Chung Hsuan Shan^1^ , Zhi Wen Chen^1 *^, Chuncheng Yang,^2^ Chandra Veer Singh^1,3*^

# **Section I: Density Functional Theory Calculations**

The Vienna ab initio simulation package (VASP) was used to perform all DFT calculations and all computations were performed on remote clusters provided by Compute Canada. The core electrons were described by a projector-augmented wave (PAW) pseudopotential and generalized gradient approximate (GGA) with a Perdew-Burke-Ernzerhof function (PBE) [1], [2]. The wave function calculations had a 550 eV kinetic energy cutoff with a Monkhorst-Pack grid of 4×4×1, a 15 Å vacuum gap on top of the material system and periodic boundary conditions. The k-points mesh was found to be sufficiently accurate based on a basic convergence test ranging from 1×1×1 gamma k-points to 5×5×1 gamma k-points. All geometric relaxations were performed with a 10^-5^ eV and 0.02 eV/Å convergence criterion for energy and forces.

The IrPdPtRhRu HEA nanoparticle surface was modeled through a Neural Generator produced by Anand et al [3]. The neural generator was used to create random 4×4×4 HEA slabs of approximate equiatomic ratios (Figure S1) [3]. The slabs were generated as 100/110/111 surfaces and a parametric sweep was conducted to find the ideal lattice parameter down to 0.01 Å accuracy. 400 unique datapoints were simulated for each of the 3 surfaces and a datapoint was only removed from the database if surface migration occurred. The 3 different surfaces were considered because they are found to be common on FCC HEA system surfaces [4].

Adsorption energy calculations were performed using a standard adsorbate/slab:

${\Delta E}_{ads}=E_{H+slab}-E_{slab}-0.5*E_{H2}$(1)

Where $\Delta$*E*_ads_ is the adsorption energy of hydrogen, *E*_H+slab_ is the total energy of a hydrogen atom adsorbed onto a HEA slab, *E*_slab_ is the total energy of the HEA slab, and *E*_H2_ is the total energy of a H_2_ molecule in vacuum. The adsorption free energy is defined in equation (2) but for the HER in acidic conditions, Norskov’s overpotential approximation can be applied to obtain the free energy of a system directly from the adsorption energy as shown in equation (3) [5].

$\Delta G_{ads}=\Delta E+\Delta ZPE-T\Delta S$(2)

$\Delta G_{ads}\approx\Delta E_{ads}+0.24$(3)

Where $\Delta G$, $\Delta E$, $\Delta ZPE, T,\Delta S$ are adsorption free energy (eV), adsorption energy (eV), zero point energy change (eV), temperature (K) and entropy change (eV/K) respectively.

High surface coverage datapoints were further analyzed with electron density, d-band positioning, and density of states (DOS) calculations. Bader charge analysis was conducted with the Henkelman groups VASP software package [6]. A higher resolution 8×8×1 KPOINTS grid was used for DOS calculations.

ML predicted HEA structures were simulated on 111 surface slabs where a random 4×4×4 slab was extracted from the database and the 15 nearest atoms to the adsorbate were replaced using with ML predicted compositions.

# **Section II: Model Design & Optimization**

Preliminary model exploration was conducted using sci-kit learn, PyTorch, and GPyTorch models to evaluate Random Forest (RF), Gradient Boosted Regression (XG), Exact Gaussian process regression (GPR), Decision Tree (DT), Linear Regression, Support Vector Machine regression (SVM), and Multi-layer-perceptron Neural Networks (NN). The GPR and NN models were ultimately chosen for further optimization. A geometric structure was converted into a model interpretable input by the pipeline shown in Figure S6. The 15 nearest atoms to an adsorbate were extracted, and a list of descriptors was applied to each atom to generate a 15×*N* array where each row represents the descriptors associated with a specific atom and each column *N_i_* is a given descriptor. The array would then be flattened in a 1D tensor where the leftmost descriptors were associated with the atoms closest to the adsorbate and the rightmost descriptors were associated with the furthest atoms.

Over 60 unique numeric descriptors were initially evaluated for each model. The descriptors were accumulated from Mendeleev’s standard element descriptor library and literature research. The importance of each descriptor was measured by taking the Pearson Correlation score of the 15×*N* descriptors relative to a datapoint’s overpotential label and then calculating the mean absolute value of each descriptor across the 15 atoms where that individual descriptor was embedded. The results are tabulated in Table S2. A combination of high impact descriptors and electronic descriptors was chosen for non-Bayesian models and a smaller set was chosen for Bayesian models. The reason of certain lower correlated electronic descriptors were selected because similar properties were considered and discussed in the experimental HEA nanoparticle system and are applicable to the computational models and therefore can ensure non-redundant, meaningful inputs for the model. There were also negligible model performance differences when using the experimental descriptors as opposed to DFT calculated values [7]. All ML performance metrics discussed in this work were after dataset shuffles, then trained and tuned at an 80/10/10 train/validation/test split ratio.

## The GPR model utilized descriptors of *N*_ied_, d-band center and atomic number. A dataset oversampling technique, Synthetic Minority Over-Sampling Technique for Regression with Gaussian Noise (SMOGN), was implemented from the smogn python module and was applied to oversample the rare overpotential ranges (Figure S7). The oversampling is done by combining techniques of k-nearest neighbors (KNN) and interpolation, and the addition of Gaussian noise for larger KNN distance region to increase diversity in the synthetic points. For exact SMOGN parameters, see Table S3. To ensure the oversampling technique was providing physically realistic predictions, the entire optimization process was compared against a benchmark NN model. For specific details on the architecture of the NN and GPR models used for the study, see section SII A and SII B.

To benchmark the descriptor-based ML models against top performing regression models, the ALIGNN graph neural network (GNN) was applied to the HER dataset [9].

## **A. Neural Network Model**

The multi-layer perceptron neural network was implemented through Pytorch. The model consisted of 3 hidden layers of 120, 24, and 12 nodes + bias with ReLu activation functions. Training, validation and testing was done with shuffled batch size of 32. The model was optimized using Adam optimizer for a mean squared error loss function(MSELoss) and was trained for 5000 epochs at a intial learning rate of 1e-4 and a reduced learning rate on plateau scheduler with default hyperparameters.

## **B. Gaussian Process Regression Model**

The GPR model used for optimization was an ExactGP model implemented through GPytorch. The mean function, covariance function and likelihood function are selected to be ConstantMean, MultivariateNormal and GaussianLikelihood respectively. A scale_to_bounds utility function was also included to the GPR model to normalizing the input between [-1,1]. Adam was selected as the optimizer, with ExactMarginalLogLikelihood as the loss function.

## **C. Performance Optimization**

The 15 nearest atoms to the adsorbate of each datapoint in the database were considered for compositional analysis. To sufficiently explore the compositional-performance space of the HEA, the ML models were tasked with predicting randomly generated surfaces in terms of the 15 nearest atoms to the adsorbate that fit within experimentally confirmed compositional criteria. Once 20,000 unique structures were predicted to possess free energies close to 0 eV (< |0.09 eV|), the structures and predicted free energies were saved for further compositional analysis.

Sample surfaces were extracted for DFT verification to confirm the ML model’s accuracy. Since the optimized surfaces did not deviate significantly from equiatomic ratios, consideration of the thermal/entropic stability of the HEA was not given because the group which published the original work on one-pot polyol synthesized IrPdPtRhRu HEAs confirmed the HEA is stable in ratios up to 6:1:1:1:1 for any of the 5 elements. All DFT verified structures were simulated on the 111 surfaces.

## **D. Techno-economic Analysis**

A techno-economic analysis of the HEA was performed to optimize cost based on chemical reagent cost, historic raw metal cost, and performance. The ML models predicted 3 sets of optimal compositions for performance. However, cost analysis revealed the large difference in synthesis cost of the two compositions. For a full visualization of the optimization workflow, see Figure S5.

# **Section III: Supplementary Figures**


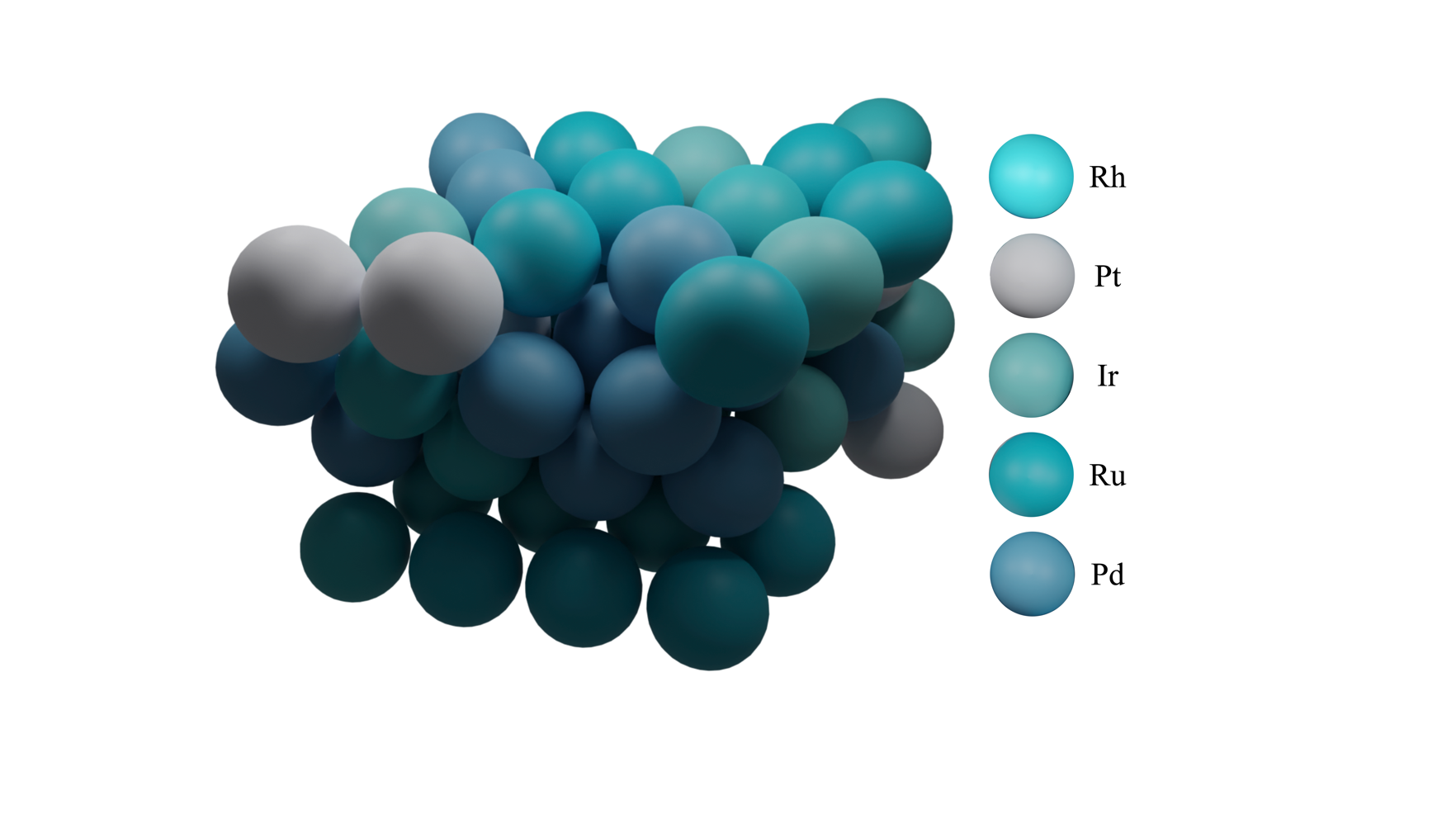


**Figure S1.** 4×4 supercell with 4 layers of IrPdPtRhRu HEA in 111 surface generated by Anand et al. Neural Generator [3].


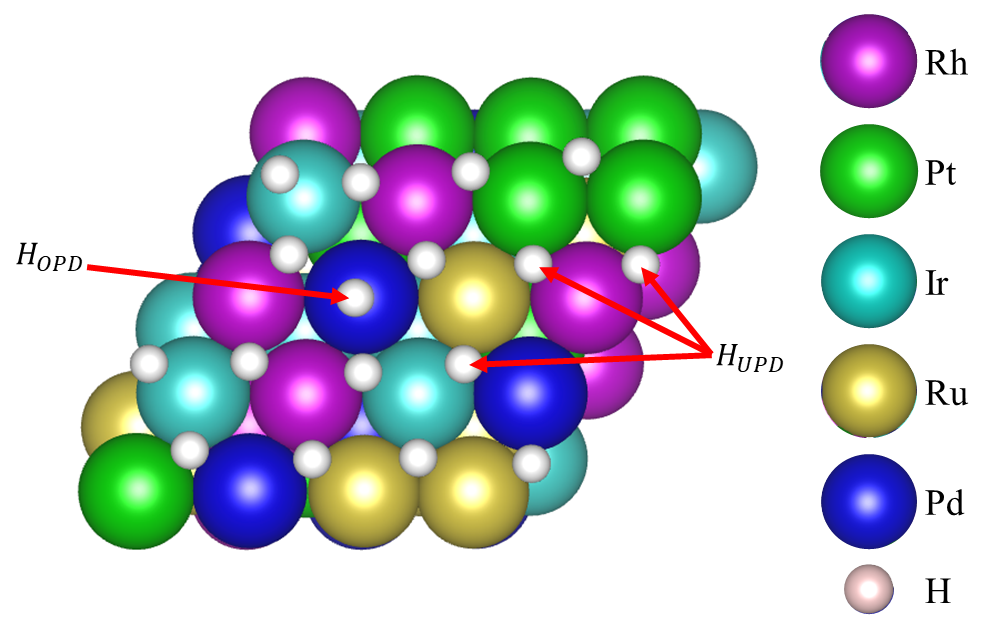


**Figure S2.** The structure of the 4×4×4 FCC HEA with 1 monolayer of H_UPD_ adsorbed in the FCC hollow sites and a single H_OPD_ adsorbed at a top site. All the unlabeled hydrogen atoms are of H_UPD_ type. The electronic asymmetry on the HEA surface causes some H_UPD_ atoms to be slightly displaced from the perfect FCC hollow site position.


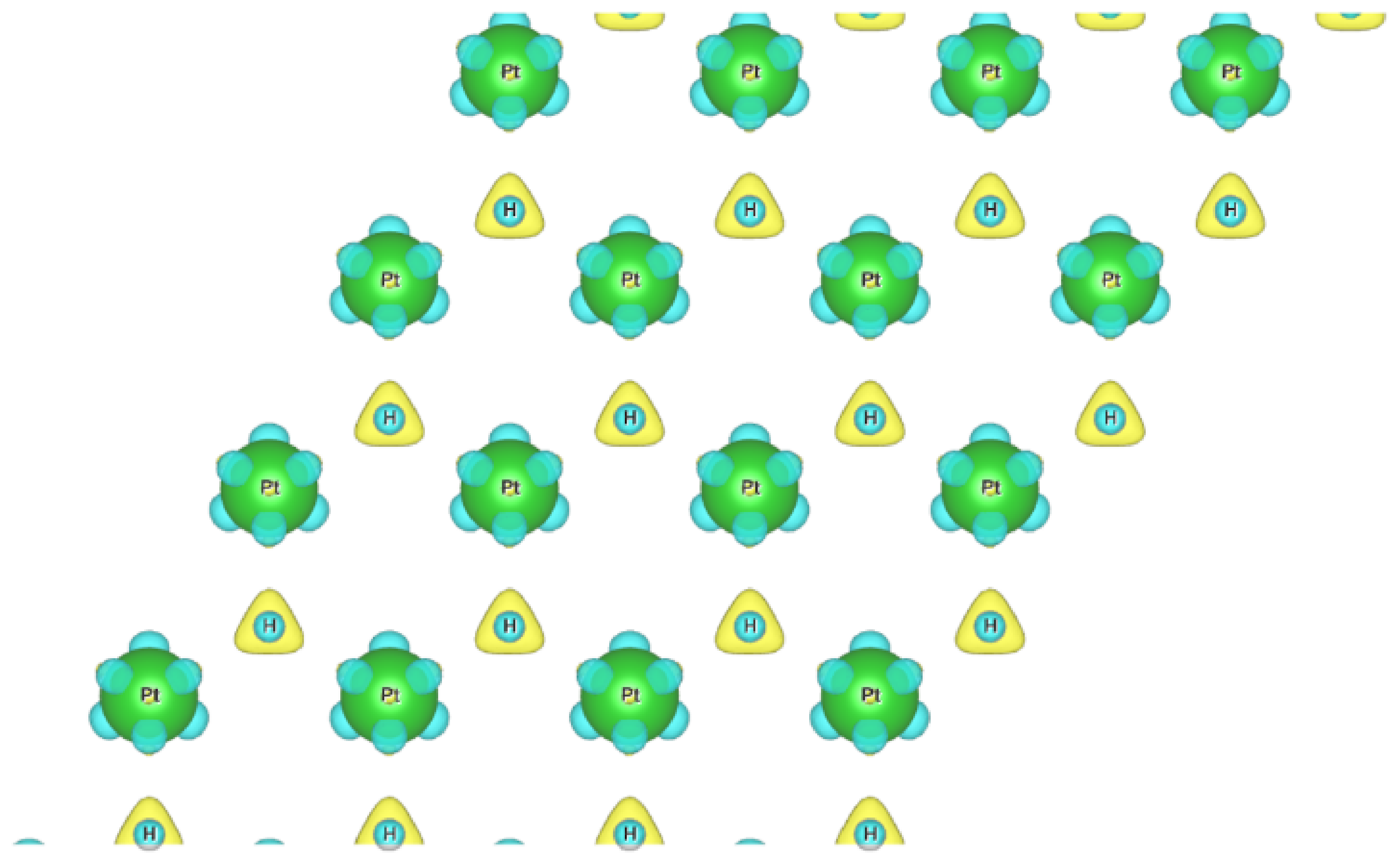


**Figure S3.** The charge density difference of 1 monolayer HUPD adsorbed on Pure Pt. The yellow isosurface represents electron accumulation and the cyan isosurface represents electron depletion with an isosurface level of 0.01.


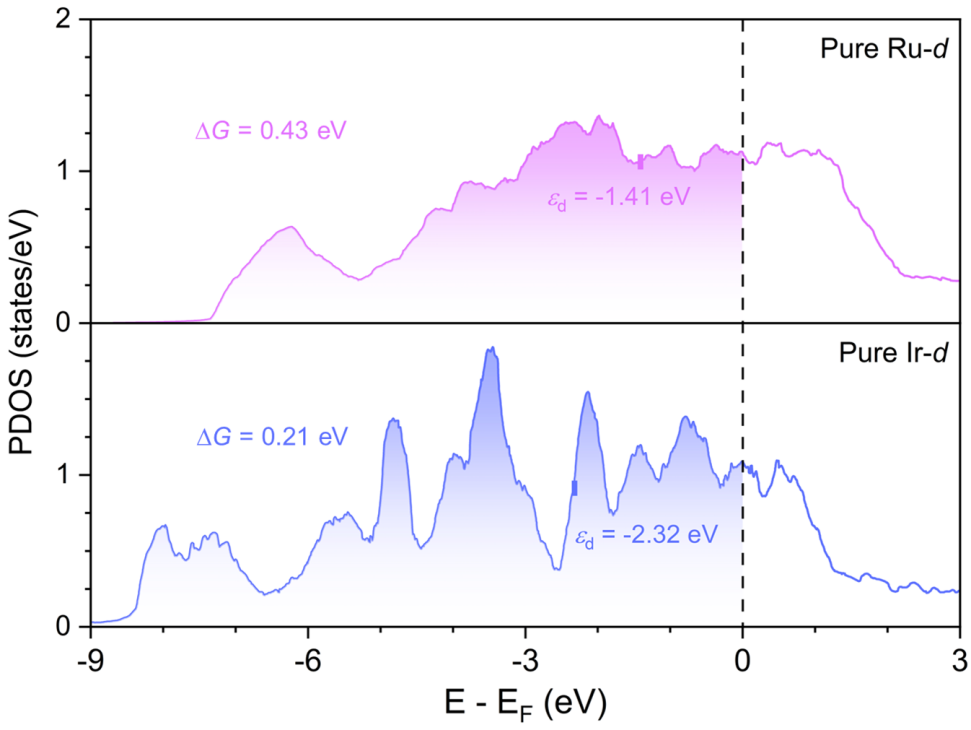


**Figure S4** The projected density of states (PDOS) for top site of pure Ru and Ir with 1ML H_UPD_.


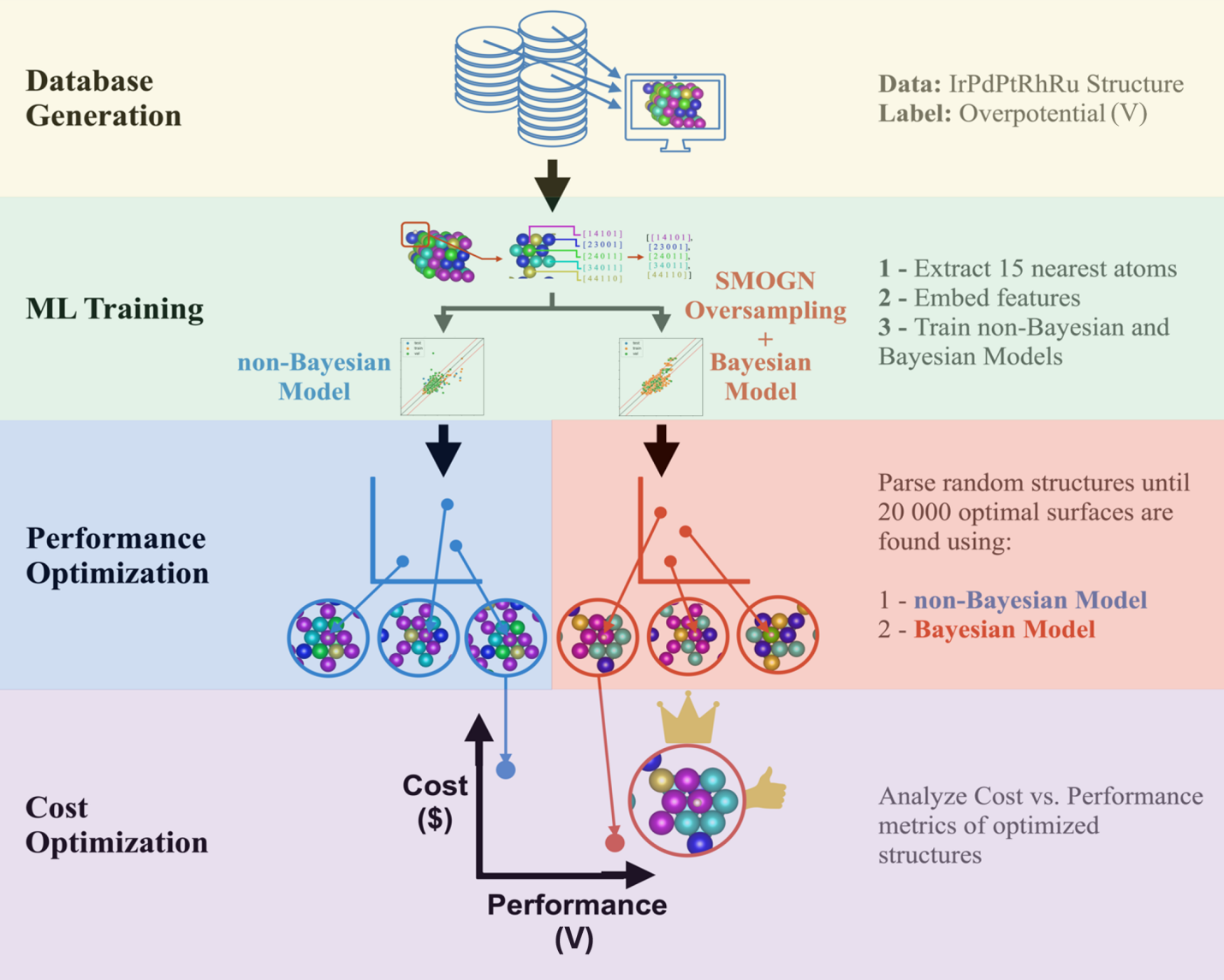


**Figure S5.** Visualization of the workflow of composition optimization for low-surface coverage scenario. Step 1 is to generate a database of IrPdPtRhRu surface configurations and calculate the overpotential values for each unique H adsorption site. Step 2 is to embed descriptors into each datapoint’s unique structure and to train a Bayesian and non-Bayesian model on the database. The non-Bayesian model is trained as a baseline reference. Step 3 is to parse random structures until both models discover 20,000 optimal overpotential structures. Step 4 is to compare the cost and performance of optimized structures to extract trends and ideal surfaces.


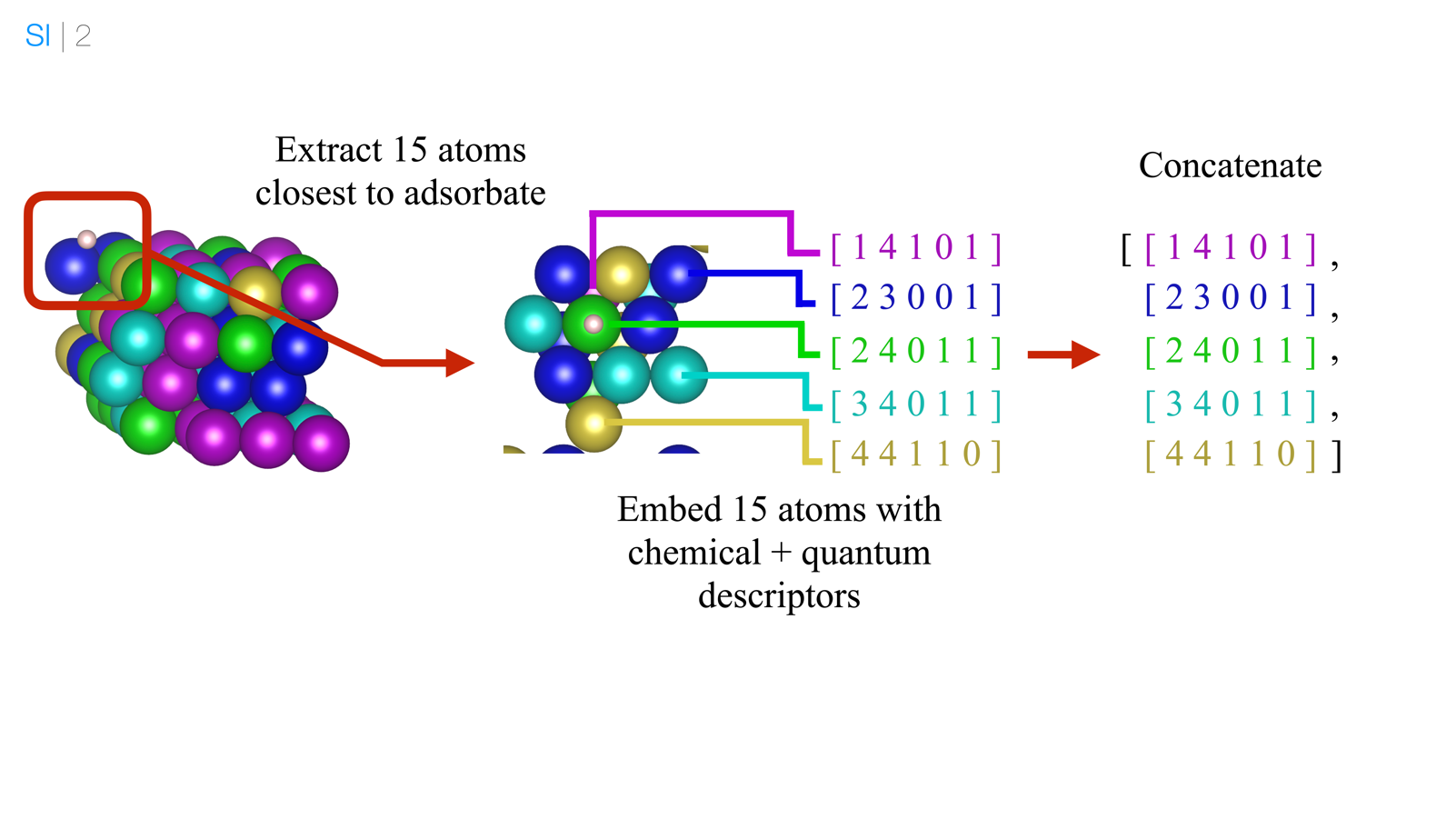


**Figure S6.** DFT CONTCAR conversion to Pytorch interpretable tensor format. Boundary Conditions considered using ASE radial functions.


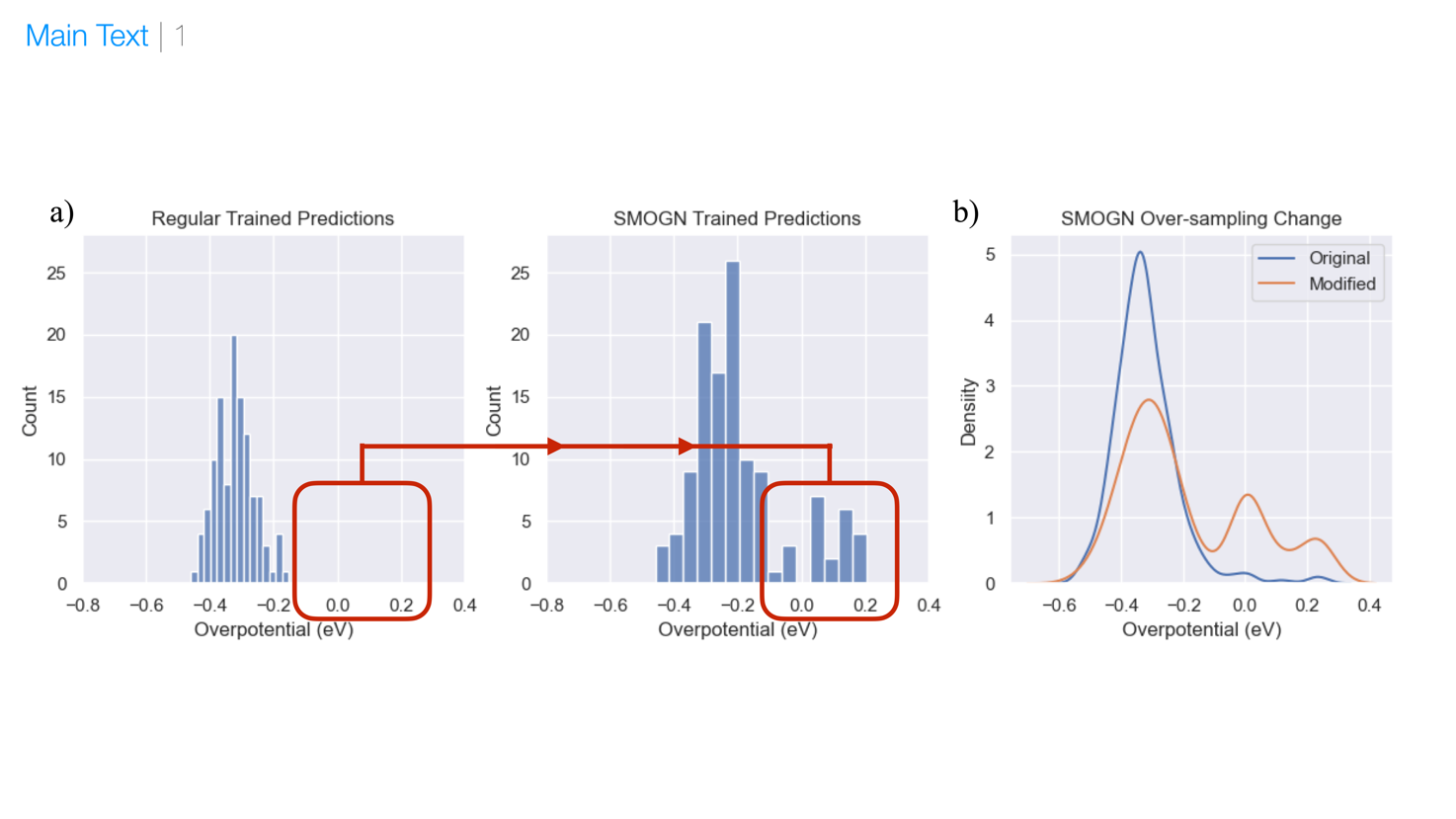


**Figure S7.** a) Overpotential distribution of test set predictions from GPR models with a tight distribution centered around –0.3 eV due to little outlier predictive capability and overpotential distribution of test set predictions from GPR models with a SMOGN oversampling applied to the dataset showing a broader distribution with improved outlier predictive capability. b) Overpotential distribution of un-modified and SMOGN modified dataset used to train models.


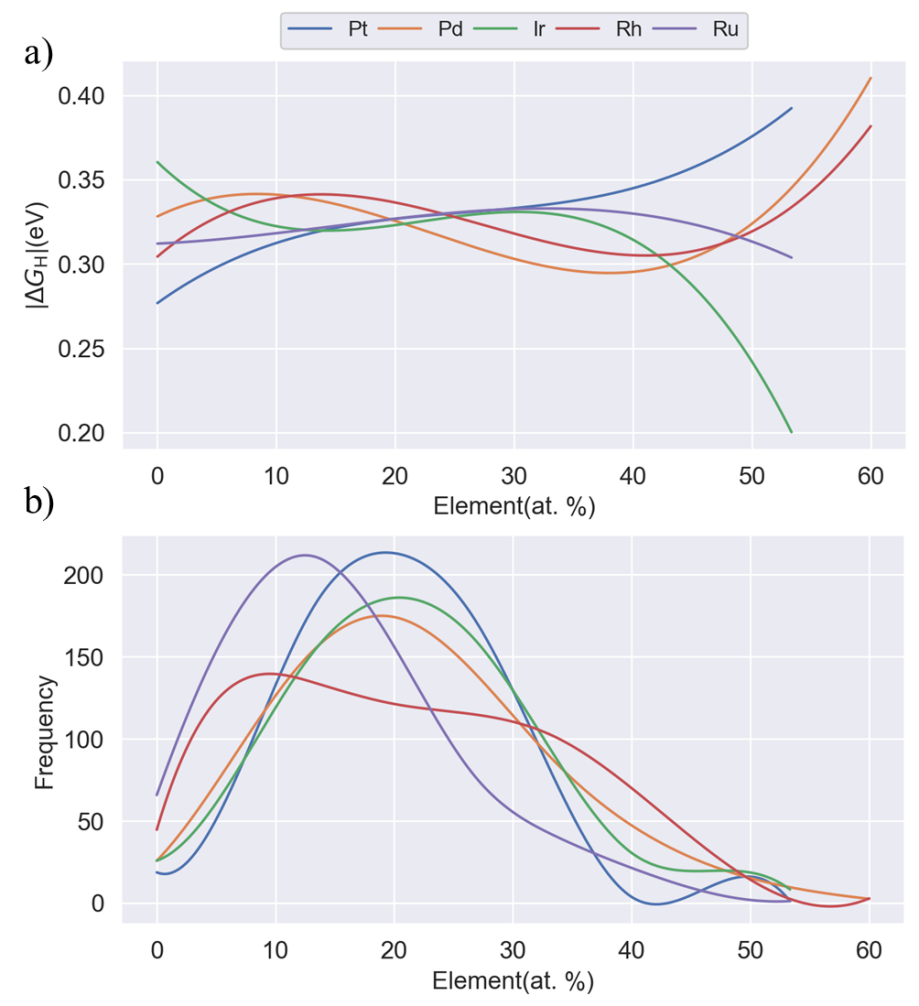


**Figure S8.** a) Average |*∆G_H_*| and b) data frequency versus atomic percentage of metal components in the low-coverage hydrogen adsorption database.


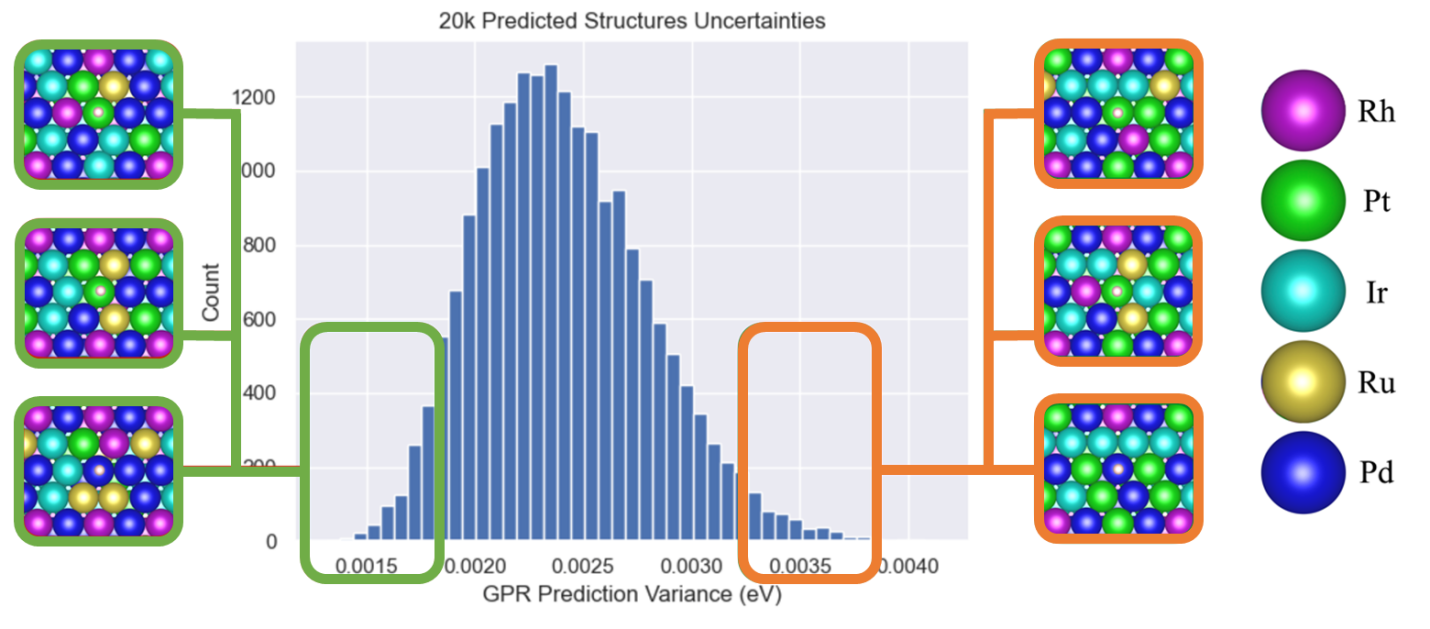


**Figure S9.** Uncertainty associated with each of the 20,000 predicted structures between –0.09-0.0 eV overpotential and the range where the highest certainty (red) and lowest certainty (green) structures were extracted from. Surfaces visualized with atomic color code consistent with all atomic visualizations.


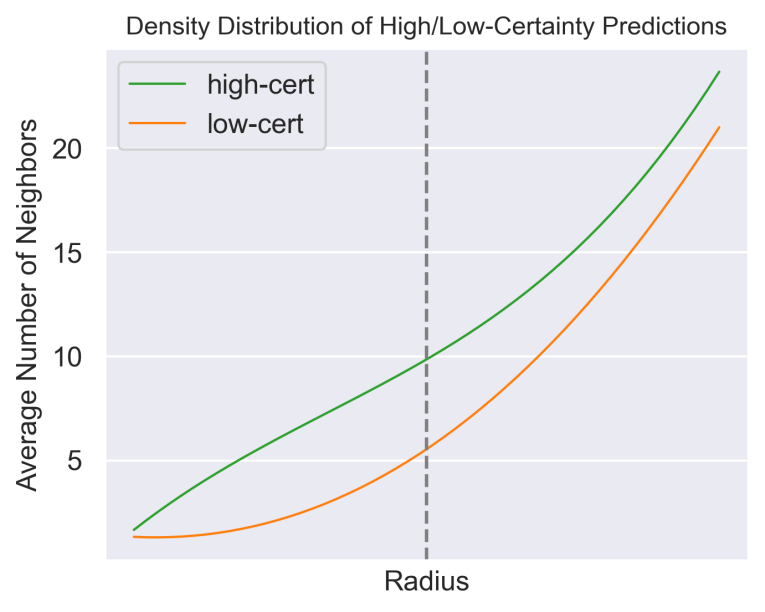


**Figure S10.** Average data density distribution of high/low-certainty predictions. At the vertical dashed line, the average number of datapoints within cutoff radius is 9.33 and 5.0 for high- and low-certainty predictions respectively.


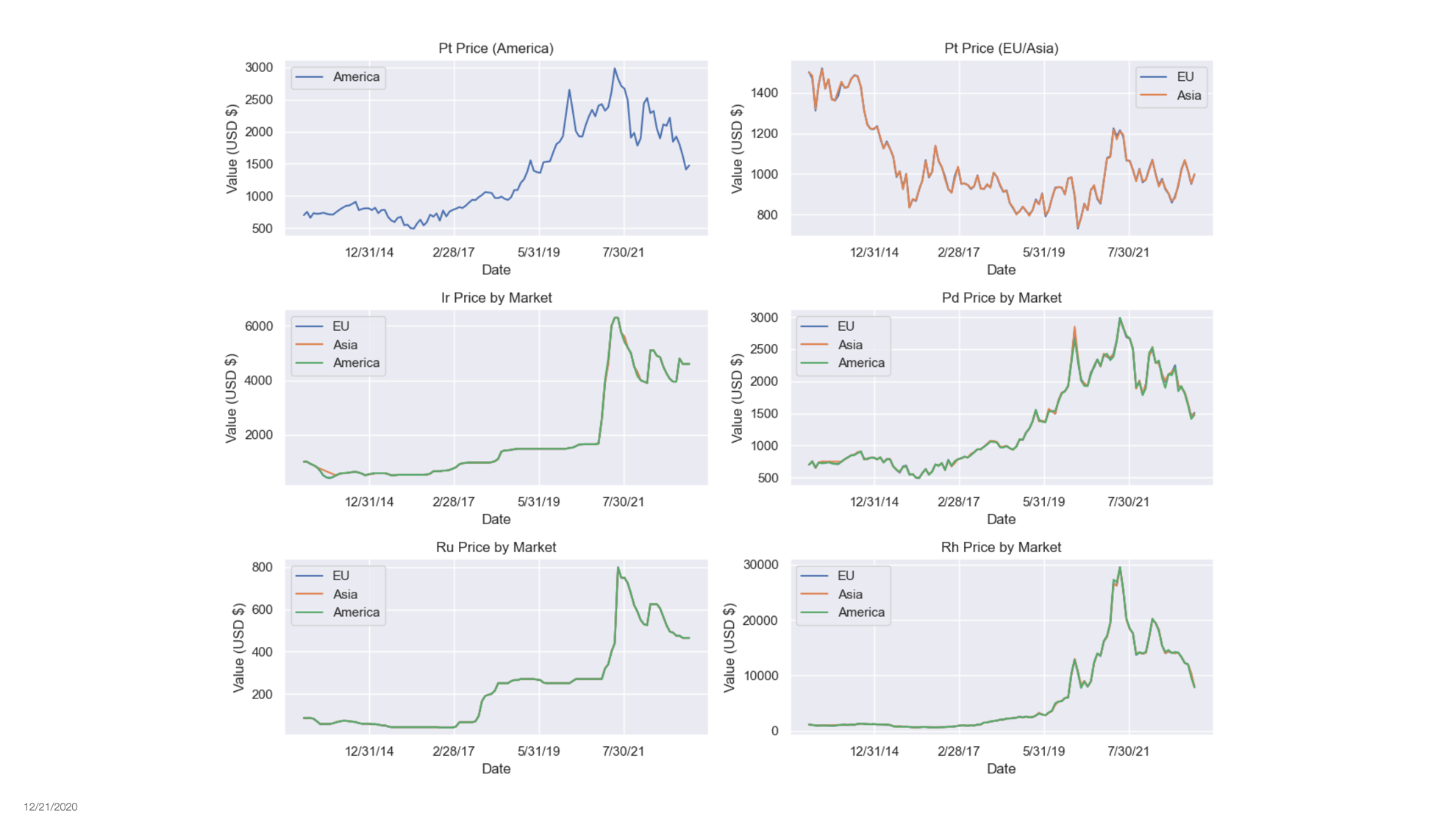


**Figure S11.** Johnson Matthey historic value of the Pt, Ir, Pd, Ru, and Rh from 2013 to 2023. Value across all 3 markets is relatively the same for every metal aside for Pt where the value varied considerably between American markets and EU/Asia markets.


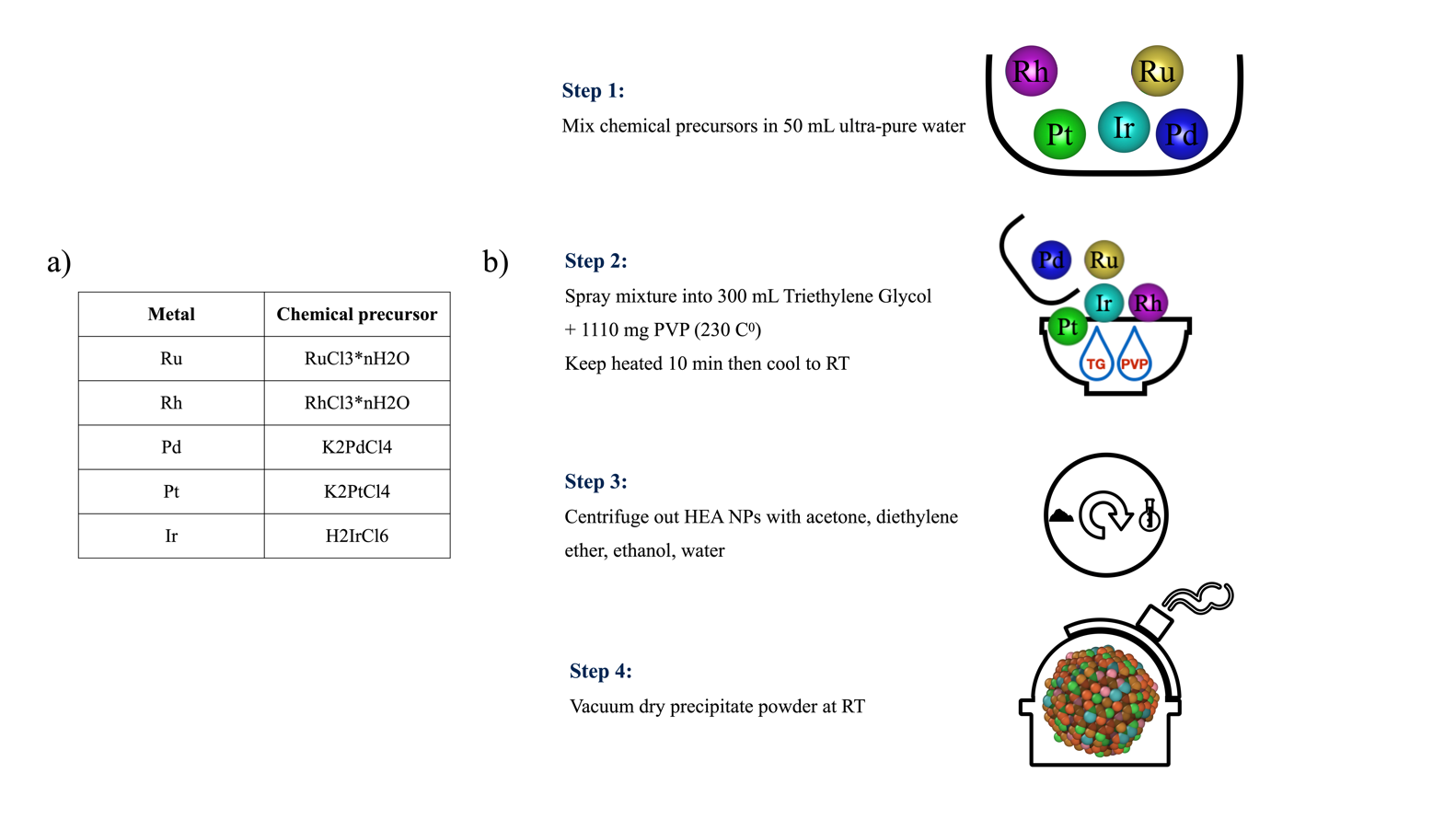


**Figure S12.** a) Chemical precursor used to add a given transition metal to the HEAs composition. b) Visualization of the 4 step one-pot polyol process to experimentally synthesize IrPdPtRhRu HEA NPs.


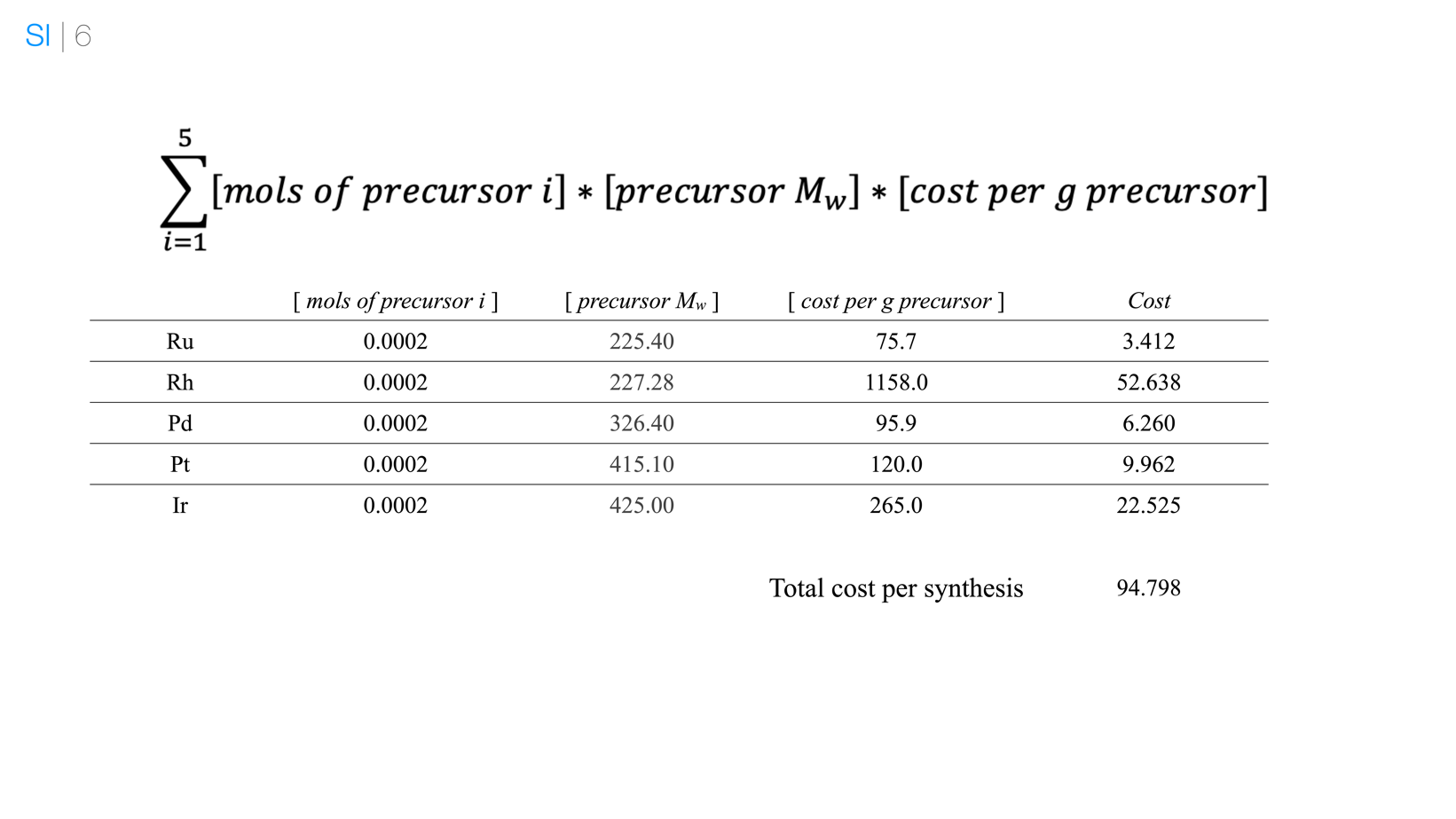


**Figure S13.** Sample calculation of cost per synthesis method used to synthesize IrPdPtRhRu HEA NPs using one-pot polyol process.

# **Section III: Supplementary Tables**

**Table S1.** *∆E_ads_* of hydrogen on pure metal surface in eV

|  | **FCC hollow** | **Atop** | **Atop** |
| --- | --- | --- | --- |
| **Metal Facet** | 1/16ML | 1/16ML | 1/16ML with the presence of 1ML FCC-hollow |
| Ru(0001) | -0.70 | -0.22 | 0.19 |
| Ir(111) | -0.48 | -0.49 | -0.033 |
| Pt(111) | -0.50 | -0.54 | 0.11 |

**Table S2.** Absolute value average Pearson Correlation score of descriptors in relation to overpotential. Each score is the absolute average of the descriptor embedded across the 15 nearest neighbor atoms over all the datapoints.

| **Absolute Average Pearson Correlation Value** | **Descriptor** | **Absolute Average Pearson Correlation Value** | **Descriptor** |
| --- | --- | --- | --- |
| 0.0404 | en_pauling | 0.0712 | metallic_radius_c12 |
| 0.0498 | Zunger d shell radii | 0.0712 | metallic_radius |
| 0.0509 | binding energy of individual element | 0.0741 | covalent_radius_cordero |
| 0.0517 | covalent_radius_pyykko_double | 0.0803 | Valency |
| 0.0529 | electron_affinity | 0.0804 | atomic_volume |
| 0.0533 | Zunger s shell radii | 0.0814 | atomic_radius |
| 0.0540 | specific_heat | 0.0839 | group_id |
| 0.0542 | vdw_radius | 0.0862 | thermal_conductivity |
| 0.0551 | period | 0.0867 | dipole_polarizability |
| 0.0555 | lattice_constant | 0.0868 | boiling_point |
| 0.0556 | en_ghosh | 0.0872 | covalent_radius_pyykko_triple |
| 0.0557 | density | 0.0872 | dipole_polarizability_unc |
| 0.0561 | atomic weight | 0.0888 | d-band width |
| 0.0561 | atomic_number | 0.0891 | Boiling points |
| 0.0578 | en_allen | 0.0904 | pettifor_number |
| 0.0580 | vdw_radius_uff | 0.0908 | heat_of_formation |
| 0.0585 | d-band center | 0.0921 | glawe_number |
| 0.0604 | Modulus | 0.0922 | vdw_radius_alvarez |
| 0.0608 | c6_gb | 0.0924 | abundance_crust |
| 0.0642 | work function | 0.0928 | fusion_heat |
| 0.0660 | vdw_radius_batsanov | 0.0933 | atomic_radius_rahm |
| 0.0661 | first ionization energy | 0.0967 | number of valence electrons in d orbitals |
| 0.0696 | covalent_radius_pyykko | 0.0967 | Nied |
| 0.0711 | Zunger p shell radii |  |  |

**Table S3.** SMOGN hyperparameters used for oversampling GPR datasets using SMOGN python package (version 0.1.2).

| **Parameter** | **Value** |
| --- | --- |
| K value (neighbor count) | 6 |
| Sampling method | balance |
| NA removal | FALSE |
| Rel threshold (rarity threshold) | 0.9 |
| Rel method | manual |
| Rel control (regions of interest) | rg_mtrx |

# **References**

[1] M. Ernzerhof and G. E. Scuseria, Assessment of the Perdew-Burke-Ernzerhof exchange-correlation functional, *Journal of Chemical Physics* **1999**, 110 (11) 5029–5036. DOI: 10.1063/1.478401

[2] J. P. ; B. K. ; E. M. Perdew, Generalized gradient approximation made simple, *Physical Review Letters* **1996**, 77 (1396) 3865-3896. DOI: 10.1103/PhysRevLett.77.3865

[3] C. Feugmo, K. Ryczko,  A. Anand, C. V. Singh, I. Tamblyn, Neural evolution structure generation: High entropy alloys, *The Journal of Chemical Physics* **2021**, 155. DOI: 10.1063/5.0049000

[4] Y. N. Wen and J. M. Zhang, Surface energy calculation of the fcc metals by using the MAEAM, *Solid State Communications* **2007**, 144 (3) 163–167. DOI:10.1016/j.ssc.2007.07.012

[5] J. K. Nørskov *et al.*, Trends in the Exchange Current for Hydrogen Evolution, *Journal of the Electrochemical Society* **2005**, 152 (3) 988-995.

[6] W. Tang, E. Sanville, and G. Henkelman, A grid-based Bader analysis algorithm without lattice bias, *Journal of Physics Condensed Matter* **2009**, 21 (84) 204-210. DOI:10.1088/0953-8984/21/8/084204

[7] B. Hammer and J. K. Nørskov, Theoretical Surface Science and Catalysis-Calculations and Concepts. *Advances in Catalysis* **2000**, 45 (45) 71-129. DOI:10.1016/S0360-0564(02)45013-4

[8] V. L. Deringer, A. P. Bartók, N. Bernstein, D. M. Wilkins, M. Ceriotti, and G. Csányi, Gaussian Process Regression for Materials and Molecules, *Chemical Reviews* **2021**, 121 (16) 10073–10141. DOI: 10.1021/acs.chemrev.1c00022

[9] K. Choudhary and B. Decost, Atomistic Line Graph Neural Network for Improved Materials Property Predictions, *NPJ Computational Materials* **2021**, 7 (185) 650-671. DOI: 10.1038/s41524-021-00650-1
